# Are Bacterial Persisters Dormant Cells Only?

**DOI:** 10.3389/fmicb.2021.708580

**Published:** 2022-02-02

**Authors:** Jin Zou, Bo Peng, Jiuxin Qu, Jun Zheng

**Affiliations:** ^1^Department of Clinical Laboratory, The Third People’s Hospital of Shenzhen, Southern University of Science and Technology, National Clinical Research Center for Infectious Diseases, Shenzhen, China; ^2^Faculty of Health Sciences, University of Macau, Zhuhai, Macau SAR, China; ^3^School of Life Sciences, Sun Yat-sen University, Guangzhou, China; ^4^Laboratory for Marine Biology and Biotechnology, Qingdao National Laboratory for Marine Science and Technology, Qingdao, China; ^5^Institute of Translational Medicine, University of Macau, Zhuhai, Macau SAR, China

**Keywords:** persister, dormancy, cell wall deficient bacteria, spheroplast, L-form

## Abstract

Bacterial persisters are a sub-population of phenotypic variants that tolerate high concentrations of antibiotics within the genetically homogeneous cells. They resume division upon the removal of drugs. Bacterial persistence is one of major causes of antibiotic treatment failure and recurrent infection. Cell dormancy, triggered by toxin/antitoxin pair, (p)ppGpp, SOS response and ATP levels, is known to be the mechanistic basis for persistence. However, recent studies have demonstrated that bacteria with active metabolism can maintain persistence by lowering intracellular antibiotic concentration via an efflux pump. Additionally, others and our work have showed that cell wall deficient bacteria (CWDB), including both L-form and spheroplasts that produced by β-lactam antibiotics, are associated with antibiotic persistence. They are not dormant cells as their cell walls have been completely damaged. In this review, we discuss the various types of persisters and highlight the contribution of non-walled bacteria on bacterial persistence.

## Introduction

Bacterial persisters refer to a subpopulation of non-growing bacteria that are able to survive the transient exposure to high concentration of an antibiotic (Balaban et al., 2019). Bacterial persisters were first described by Joseph Bigger in 1944, who found a small sub-population of *Staphylococci* survived from the killing by penicillin. These bacterial survivors subsequently recovered growth and maintained antibiotic susceptibility after removal of the drug (Bigger, 1944). Harris Moyed found that the persistence associated with a toxin and anti-toxin system (Moyed and Bertrand, 1983). Subsequently, Balaban et al. (2004) confirmed the survival of persisters using microfluidic devices and live imaging. Unlike the heritable resistance, bacterial persistence is a trait found only in a small fraction of cells that are isogenic, and thus are the phenotypic variants of the bacterial population produced stochastically or induced by environmental factors (Harms et al., 2016). In recent decades, additional to antibiotic resistance, persisters were recognized as another major cause of the antibiotic treatment failure and recalcitrance of bacterial infections (Hall-Stoodley et al., 2004; Harms et al., 2016; Gollan et al., 2019), and considered as the main reason for the antibiotic tolerance of biofilms (Lewis, 2001; Spoering and Lewis, 2001; Yan and Bassler, 2019). Importantly, persistence is believed to facilitate and promote the evolution and emergence of resistance (Cohen et al., 2013; Levin-Reisman et al., 2017; Windels et al., 2019; Liu J. et al., 2020).

In the past 20 years, the understanding on the mechanistic formation of persistence has made extensive advances. Cell dormancy is considered as the fundamental mechanistic basis of persister formation: bacterial persisters are in a dormant state, and the pathways containing the drug target in such dormant cells are inactive, and thus antibiotics cannot exert their lethal effects even they successfully bind to their targets. The bacterial cells undergone antibiotic treatment therefore are not damaged and maintain an intact cellular structure (Fisher et al., 2017). However, recent study suggested that bacterial persisters from ofloxacin treatment originated from metabolically active cells (Goormaghtigh and Van Melderen, 2019). Other studies also showed that bacteria can actively employ efflux pumps to facilitate persistence formation (Pu et al., 2016, 2017). The studies suggested that such bacterial persisters are not passive dormant cells. This notion was further reinforced by several other works showing that antibiotic treatment generated the non-walled bacterial cells which form an important type of persisters (Cross et al., 2019; Mickiewicz et al., 2019; Zou et al., 2020). In this review, we will briefly introduce the dormant persisters and attempt to discuss the non-walled bacteria as the bacterial persisters.

## Bacterial Persistence by Cell Dormancy

**FIGURE 1:**
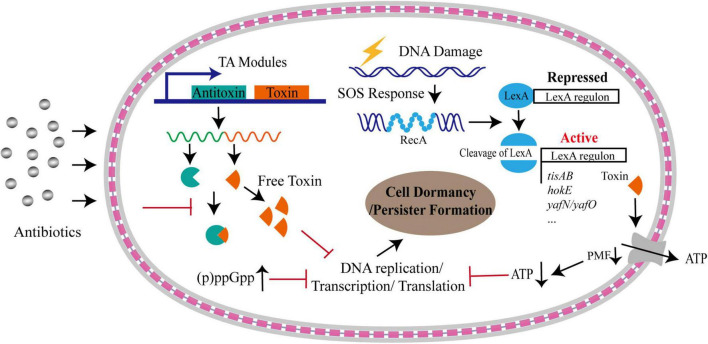
Formation of bacterial persistence via cell dormancy. Various pathways could trigger bacterial cells into dormant state upon antibiotic stress. A considerable number of TA modules could free toxins upon stresses, which result in persister formation by inhibiting DNA replication, or transcription, or translation processes, or downregulating proton motive force (PMF) that is under the control of SOS response. Dormancy could be also triggered by accumulated alarmone molecules and decreased intracellular ATP levels. The gray layers represent the outer membrane and inner membrane, respectively, and the red square linkage represents the peptidoglycan and periplasmic space.

Several mechanisms could trigger the cells to enter the dormancy, including toxin-antitoxin (TA) system (Balaban et al., 2004; Keren et al., 2004; Lewis, 2007; Dorr et al., 2010), alarmone molecule, SOS response and intracellular ATP level (Dorr et al., 2009; Shan et al., 2017; Figure 1).

### (p)ppGpp

The alarmone (p)ppGpp includes a guanosine pentaphosphate and guanosine tetraphosphate. It is synthesized and degraded mainly by RelA-SpoT homolog (RSH) family enzymes (Hauryliuk et al., 2015). This alarmone can be induced and accumulated during stringent response, including responses to amino acid starvation or other nutrition limitations, such as fatty acids, carbon and nitrogen (Gaca et al., 2015; Brown, 2019). For example, in response to nutrient starvation, GTPase Obg-mediated persistence in *E. coli* required (p)ppGpp to activate the transcriptional expression of type I HokB-SokB TA module. Finally, the elevated levels of HokB toxin resulted in membrane depolarization, leading to cell dormancy (Verstraeten et al., 2015). Upon production, (p)ppGpp also directly inhibits RNA polymerase or several enzymes involved in translation process as well as GTP biosynthesis, resulting in the translation repression and growth arrest that facilitate bacterial survival under various environmental stress (Magnusson et al., 2005; Hauryliuk et al., 2015; Figure 1). By systematical examination on the relationship of 15 common persister genes with (p)ppGpp, Liu et al. (2017) identified that two genes, namely *dnaK* and *recA*, were implicated in persistence to ampicillin and gentamicin and the persistence level dependent on (p)ppGpp,while other genes exhibited various relationship with (p)ppGpp. In addition, they found that the persister gene interaction with (p)ppGpp was affected by culture age, cell concentrations and antibiotics. Despite the elevated levels of (p)ppGpp plays an important role in the formation of persisters (Korch et al., 2003; Svenningsen et al., 2019), the detailed mechanism mediated by (p)ppGpp needs to be further investigated.

### Toxin-Antitoxin Systems

Toxin-antitoxin systems are ubiquitous modules identified in almost all bacteria. The systems consist of a toxin and a corresponding antitoxin. The toxin is an element that is able to inhibit diversely important cellular processes, including cell wall synthesis, ATP synthesis, protein translation and DNA replication, and the antitoxin can prevent the function of the cognate toxin (Yamaguchi et al., 2011; Hall et al., 2017). Toxin of the TA system is typically a protein, whereas the antitoxin is diverse, and can be a non-coding RNA in type I and III TA systems, or a protein in the other TA systems (Page and Peti, 2016; Hall et al., 2017). Till today, seven different types of TA systems, namely type I–VII, have been identified according to the nature of antitoxins and their various mechanisms of action (Yamaguchi et al., 2011; Wang et al., 2021).

*hipA* (for high persistence A) is the first toxin gene in *Escherichia coli* K-12 that associated with bacterial persistence identified by Harris Moyed when he screened for the mutants with higher frequency of persistence to ampicillin treatment (Moyed and Bertrand, 1983). The identified high-persistence allele, *hipA7*, increased the frequency of persistence by 10,000 folds (Moyed and Bertrand, 1983; Moyed and Broderick, 1986; Scherrer and Moyed, 1988). Characterization of *hipA7* allele revealed two mutations (G22S and D291A) that confer the increased persistence via diminishing the binding of HipA to HipB and the affinity of HipBA for its own operator, thus leading to increased HipA to trigger cell dormancy (Schumacher et al., 2015). It is now known that HipA is the toxin of the type II TA system HipBA, and functions as a protein kinase that inhibits cell growth by phosphorylating the glutamyl-tRNA synthetase at the conserved Ser^239^ site (Germain et al., 2013). Overexpression of HipA produces a high frequency of persister cells, and deletion of HipBA causes a decrease in persister formation in both stationary and biofilm cells in the presence of mitomycin C or ciprofloxacin (Lewis, 2005; Korch and Hill, 2006). The toxic effects on cell growth caused by HipA can be neutralized by the antitoxin HipB through forming the high order HipA-HipB-DNA complexes, which obstructs the active sites of HipA, leading to its inactivation (Black et al., 1994; Schumacher et al., 2009). However, it is worthy to note that several studies shown that deletion of HipBA did not affect the frequency of persister cells under other conditions (Black et al., 1994; Luidalepp et al., 2011). These controversial results may suggest that the contribution of HipBA to persister formation needs further studies.

Additional to HipAB TA system, several other TA systems also contribute to the formation of bacterial persisters and their tolerances to various stresses (Tripathi et al., 2014; Hu et al., 2015; Kamruzzaman and Iredell, 2019; Ma et al., 2019; Ronneau and Helaine, 2019; Masuda et al., 2020). For instance, ectopic expression of YafQ, the toxin of YafQ-DinJ module, augmented persistence by reducing tryptophanase production which converts tryptophan into indole (Hu et al., 2015), and single-gene deletion of this module increased persister fraction and enhanced the tolerance to heat by about 10-fold (Masuda et al., 2020). Interestingly, elevated production of GhoT, the toxin of GhoT/GhoS type V TA system, increased persistence upon oxidative stress controlled by MqsR/MqsA type II TA (Wang et al., 2013). MazEF, another type II TA system, mediates growth arrest and persister formation in response to antibiotic treatment, resulting in the increase in bacterial survival (Tripathi et al., 2014). Bacteria, including the pathogenic bacteria, carries abundant TA systems in their genomes. For example, *Mycobacterium tuberculosis* encodes about 88 putative TA loci in the genome, and some of them have been proven to contribute to the increased persistence (Ramage et al., 2009; Han et al., 2010; Sala et al., 2014). A type I RNA antitoxin, SprF1 in *Staphylococcus aureus*, enhanced persister cell formation by binding ribosomes and then attenuating translation process under hyperosmotic stress (Pinel-Marie et al., 2021). *S. aureus* encodes 67 putative TA loci (Habib et al., 2018) and the TA system SprG/SprF has been shown to associate with the induction of cell stasis during internalization in human macrophages (Riffaud et al., 2019), suggesting that TA system significantly contribute to bacterial persistence during their infections *in vivo*.

### SOS Response

The SOS response is a conserved DNA repairing and regulatory mechanism, triggered by single-stranded DNA (ssDNA) originated from double-strand breaks during replication fork stalling, replication-transcription collisions and transcription stalling, or DNA damaging agents, such as UV irradiation, antibiotics, oxidative agents, and high external pressure (Baharoglu and Mazel, 2014). The induction mechanism of SOS response has been well-understood. In brief, RecA is recruited by RecBCD or RecFOR onto ssDNA to form RecA filaments, which trigger the autocatalytic cleavage of LexA. The inactivation of LexA results in derepression of the SOS regulon (Courcelle and Hanawalt, 2001). Importantly, the SOS response not only triggers the repairment of damaged DNA, but also causes changes in genome plasticity and gene expression, inducing bacterial persistence under harsh environments (Baharoglu and Mazel, 2014; Figure 1). It has been shown that, upon sub-minimum inhibitory concentration (sub-MIC) of ciprofloxacin treatment, the majority of persister cells formed in a manner dependent on the SOS response, rather than being pre-existing or produced in stochastic manner (Dorr et al., 2009). The dissociation rate of LexA from DNA targets orchestrates the bacterial SOS response, and the activity of LexA directly modifies the formation frequency of bacterial persistent cells produced by DNA damage (Butala et al., 2011). Additionally, SOS response induces several genes of TA modules in *E. coli* through the LexA regulon, such as *hokE*, *yafN/yafO*, and *tisAB/istR* (Pedersen and Gerdes, 1999; Fernandez De Henestrosa et al., 2000; Vogel et al., 2004; Singletary et al., 2009). The TisB toxin induced by ciprofloxacin-mediated SOS response resulted in the decrease in proton motive force and ATP levels, leading to the cell dormancy and multi-drug tolerance (Dorr et al., 2010; Figure 1).

### Intracellular ATP Levels

Although numerous studies point to the central role of TA systems and (p)ppGpp in the formation of bacterial persistence, they may not be responsible for the persistence in all scenario. The study on *S. aureus* found that deletion of all TA modules and ppGpp synthases had no effect on persister formation under various growth phase or antibiotic treatment (Conlon et al., 2016). Instead, the persisters were produced by stochastically entering into the stationary phase accompanied by the decrease in the intracellular ATP level, suggesting that loss in energy production induces persister formation and drug tolerance (Conlon et al., 2016; Figure 1). Consistently, the inactivation of a potassium transporter system (Kdp-ATPase) in *Mycobacterium marinum* was shown to reduce the fraction of persister formation in bacteria exposed to rifampicin by the increased intracellular ATP levels (Liu X. et al., 2020). Accumulated evidence have demonstrated that intracellular ATP level plays critical contribution to the multidrug tolerance (Manuse et al., 2021). Using a persister reporter *rrnB P1* with fluorescence-activated cell sorting (FACS), Shan et al. (2017) demonstrated that upon fluoroquinolones treatment lowering ATP levels promoted persister formation in *E. coli* by inhibiting translation process and the activity of drug targets (Figure 1). This process is independent of TA expression and (p)ppGpp regulation induced by stresses (Shan et al., 2017). Decreased ATP levels could promote the formation of protein aggresome (Pu et al., 2019). The protein aggresome was thought to be an indicator of bacterial dormancy depth and its clearance was required for persister cells to resuscitate and regrowth (Pu et al., 2019). Taken together, the control of intracellular ATP level is a potentially general mechanism for bacterial persistence.

## Bacterial Persistence by Active Efflux Pumps

**FIGURE 2:**
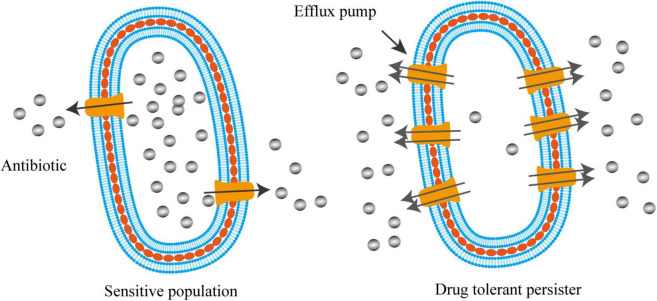
Activated efflux systems contribute to bacterial persistence. Different from cell dormancy, bacterial persisters can also employ efflux pumps with enhanced activities to defend antibiotic killing by exporting intracellular antibiotics, resulting in lower intracellular concentration of drugs that enable bacteria to survive.

Efflux systems were well-known to contribute to the drug resistance of various bacterial species. Gene inactivation showed that the lack of efflux pumps rendered mutants hyper-susceptible to multiple antimicrobial agents (Li et al., 1994, 2004; Ramon-Garcia et al., 2009). For instance, deletion of *lfrA*, a gene encoding the first reported multidrug efflux pump in *Mycobacterium smegmatis*, led to the decrease in MICs by 2–8-fold to fluoroquinolones and acriflavine (Li et al., 2004). MIC determination and time-dependent killing study also showed that carbonyl cyanide 3-chlorophenylhydrazone (CCCP), an efflux pump inhibitor disrupting membrane potential, was able to rescue the colistin susceptibility in a number of intrinsically colistin resistant bacteria, such as *Proteus* spp., *Serratia marcescens*, *Morganella morganii*, and *Providencia* spp. (Baron and Rolain, 2018). Recent work indicated that active efflux system also contributed to the formation of persister cells (Pu et al., 2016, 2017; Figure 2). Using single-cell fluorescence microscopy combined with transcriptome analysis, Pu et al. (2016) revealed that TolC, an energy-dependent outer-membrane protein of efflux family, promoted bacterial persistence in *E. coli* by rapidly exporting the antibiotic molecules, leading to a substantially lower intracellular concentration of drugs accumulated in persisters than that in the sensitive populations (Figure 2). The mechanism of persisters formation through stochastic induction of TolC pumping suggests that bacteria could use an active defense strategy, rather than the well-known passive cell dormancy, for persistence (Du Toit, 2016; Gerdes and Semsey, 2016; Pu et al., 2017). Additionally, using the *in vivo* model of *Mycobacterium marinum*-infected zebrafish, Adams et al. (2011) demonstrated that the efflux pumps of *Mycobacterium* induced by macrophage infection not only promoted intracellular survival, but also mediated drug tolerance. Supportively, proteomic analysis on persisters and non-persisters found that a number of membrane transport proteins were up-regulated in persisters (Sulaiman et al., 2018).

## Antibiotic Tolerance by Forming Cell Wall Deficient Bacteria

**FIGURE 3:**
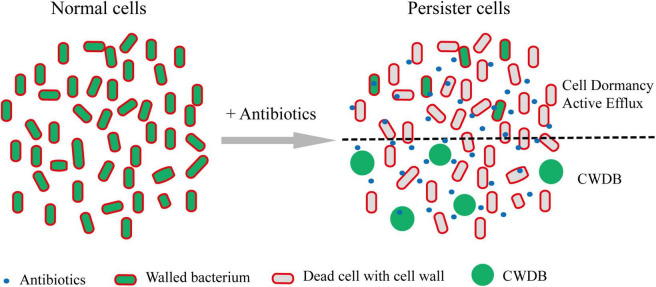
Persisters formation through CWDB. Upon antibiotic treatment, a portion of persisters maintain intact cellular structure through forming dormancy and/or active efflux pumps. In addition, some bacteria could survive after their cell walls being damaged and form spheroplast or L-form. Through an unknown mechanism, such bacteria could persist *in vivo* or *in vitro*. Persister cells with intact cell structure or cell wall deficiency are separated by black dot line.

Regardless the persistence caused by cell dormancy or active efflux pump, bacterial persisters maintained their intact cellular structure. However, recent work showed that cell wall deficient bacteria (CWDB) might be another form of bacterial persisters (Figure 3). The bacterial cell wall is a rigid, mesh-like complex structure surrounding the cytoplasmic membrane in most bacteria. It is mainly made of polysaccharide strands cross-linked by short peptides, called peptidoglycan (PG) (Vollmer et al., 2008). The main function of PG is to maintain the cell shape and structure integrity and withstand environmental osmotic forces from burst (Huang et al., 2008). Due to the critical role of cell wall in almost all bacteria and its absence in eukaryotic cells, hundreds of antibiotics, including glycopeptides, lipopeptides, and β-lactams, have been developed by targeting the cellular pathway of PG biosynthesis and cross-linking (Schneider and Sahl, 2010). Bacteria without cell wall would be quickly lysed due to the osmotic pressure. However, some bacteria develop special mechanism to overcome the osmotic pressure and survive without cell wall. The CWDB include “L-form” bacteria and spheroplast.

### “L-Form” Bacteria Associated Antibiotic Tolerance

The term “L-form” bacteria were first reported in 1935 by Emmy Klieneberger who discovered pleomorphic organisms when she attempted to isolate the pleuropneumonia-like organisms from rats’ blood (Klieneberger, 1935). These pleomorphic bacteria were later proved by Dienes to be the derivative from bacilli of *Streptobacillus moniliformis* (Dienes, 1939). Subsequently, Dienes successfully isolated L-form variants from other bacterial species and observed their reversions into the parent-form bacilli (Dienes, 1947, 1948). At present, L-forms refers to CWDB that are normally coated with cell-wall, and the L-form bacteria can only survive and proliferate with osmotic supportive media, and switch back to the walled state under appropriate culture conditions (Errington, 2013). A wide range of bacteria, either Gram-negative or Gram-positive, is able to enter into the L-form state in the presence of inhibitors of cell wall synthesis on osmoprotective media (Domingue and Woody, 1997; Errington, 2013; Figure 3). Importantly, Kawai et al. (2018) recently reported that two Gram-positive bacteria, namely *B. subtilis* and *S. aureus*, were induced L-form formation during intracellular infection by host lysozyme or other immune effectors to protect bacteria from β-lactam killing. Two classes of genetic changes were shown to be required for L-form growth in *Bacillus subtilis.* Class I mutations support L-form proliferation by driving excess membrane synthesis, which includes overexpressing of *accDA*, altogether encoding the carboxyltransferase subunit of acetyl coenzyme A (CoA) carboxylase, and the inhibition of PG precursor pathway (Mercier et al., 2013). Class II mutations promote L-form growth by reducing reactive oxygen species (ROS) damage. Most of the mutations occur in or near the *ispA*, which encodes geranylgeranyl pyrophosphate synthase in the isoprenoid synthetic pathway, resulted in the inhibition of electron transport chain activity (Julsing et al., 2007; Leaver et al., 2009; Kawai et al., 2015). Different from normal bacterial cells, the division of L-form is independent of the FtsZ-dependent machine but occurs by extrusion and resolution mechanism (Leaver et al., 2009). Due to the deficiency in cell wall, L-forms are insensitive to a range of antibiotics that target cell wall (Barbuti, 1976; Domingue and Woody, 1997). L-forms of *M. tuberculosis* showed phenotypic resistance to high concentrations of ethambutol (EMB) compared to the sensitivity of the parental strain (Slavchev et al., 2016).

### Spheroplast Associated Antibiotic Tolerance and Persistence

Spheroplast is coined to describe the spherical shape of Gram-negative bacteria with partial or complete lack of cell wall through mechanic or enzymatic method (Sun et al., 2014). spheroplast has been employed for studies in various fields, such as the properties of cytoplasmic membrane, iron channels via patch clamp analysis, and characterization of antimicrobial peptides (Martinac et al., 1987; Sun et al., 2014, 2016; Wei et al., 2016). The association of spheroplast with bacterial persistence emerged only recently. Dorr and his colleague firstly showed that *Vibrio cholerae* could form spherical non-walled cell upon a wide variety of cell wall- acting antibiotics, and the resultant cells became tolerant to these antibiotics and survived in culture media (Dorr et al., 2015). The formation of spherical antibiotic-tolerant bacterial cells was not only observed in *V. cholerae* but also in several other bacterial pathogens, including *Acinetobacter baumannii*, *Enterobacter cloacae*, *Klebsiella aerogenes*, *Klebsiella pneumoniae*, and *Pseudomonas aeruginosa* (Monahan et al., 2014; Dorr et al., 2015; Cross et al., 2019; Zou et al., 2020).

In our recent study, we investigated the morphologies of persisters in *A. baumannii* upon treatment with different classes of antibiotics, including β-lactam fluoroquinolone, and aminoglycoside. We found that a fraction of enlarged spherical cells constitutes a major sub-population of bacterial survivors from β-lactam antibiotics treatment (Zou et al., 2020). With the aid of the fluorescent D-amino acid analog HCC-amino-D-alanine (HADA) to label the peptidoglycan (PG) of the cell walls, these bacteria were shown to completely lose their cell walls upon β-lactam antibiotics treatment (Zou et al., 2020). Without the protection by cell wall, the volume of the non-walled bacteria was significantly increased (Zou et al., 2020). The non-walled cells are *bona fide* persisters but not the viable-but-non-culturable cells (VBNCs) as the subsequent time-lapse microscopy revealed that such non-walled spheroplasts survived at least for 7.5 h in the culture media and resuscitated and started to divide upon the removal of drugs (Zou et al., 2020). The non-walled spheroplast bacteria were not only observed *in vitro* in the culture media but also *in vivo* in the animal model (Zou et al., 2020). By using a strain expressing GFP reporter in a *Galleria mellonella* model, we demonstrated that β-lactam antibiotics therapy on the infected animal promoted the formation of spherical *A. baumannii* persisters *in vivo* (Zou et al., 2020).

The non-walled spheroplast bacteria is distinct from the “L-form” bacteria as the “L-form” bacteria were mostly achieved artificially by growing them on osmoprotective media in the presence of high levels of cell-wall acting antibiotics (Errington et al., 2016; Kawai et al., 2018). In contrast, the non-walled spheroplast bacteria survive in the media without any osmoprotective agent. The cell proliferation was observed for L-form bacteria but not for spheroplast bacteria (Leaver et al., 2009; Zou et al., 2020). As these non-walled spheroplasts have lost the cell wall, they presumably have an active metabolism and not in dormant state during the β-lactam antibiotics treatment (Zou et al., 2020).

### The Clinical Significance of Cell Wall Deficient Bacteria and Therapeutic Implication

Cell wall deficient bacteria, regardless L-form bacteria or spheroplast, have been reported to associate with a wide range of recurrent infection diseases (Table 1; Onwuamaegbu et al., 2005). Urinary tract infections (UTI) are commonly caused by uropathogenic *E. coli* (UPEC), especially in women and elderly, and the recurrence of infection is a major problem due to the persistence of this pathogen in the epithelium of the bladder (Brumbaugh and Mobley, 2012). A recent work demonstrated that cell wall deficient state of UPEC frequently presented in fresh urine of elderly patients suffering with recurrent urinary tract infection, and CWDB induced by antibiotics in urine were able to switch back into walled state upon the removal of drugs, which suggests the possibility of forming CWDB as a route to evade antibiotics in the recurrent infections *in vivo* (Mickiewicz et al., 2019). Similar to UPEC, *Staphylococcus aureus* is a major pathogen responsible for community- and hospital-acquired infections (Schito, 2006). It is the causes for various diseases, such as dermatitis, mastitis, toxemia, and sepsis. Animal infection model experiments suggested that cell wall deficient *S. aureus* persisters possessed the ability to produce incompetent phagocytosis by alveolar macrophages and led to the latent and chronic lung infections (Michailova et al., 2007). In addition, the transformation process of *M. tuberculosis* to CWDB was observed in clinically drug resistant isolates from patients, indicating the association of cell-wall deficient state with antibiotic resistance (Michailova et al., 2005). Despite accumulated clinical reports and experimental analyses suggested that CWDB might be involved in the pathogenesis and chronic recurrent infections, the detailed mechanism underlying the formation and switch regulation of non-walled persisters need to be further investigated.

**TABLE 1:** Cell wall-deficient bacteria associated with chronic diseases.

Cell type	Bacteria	Isolated from patients	References
L-form	*Nocardia*	Chronic mycetoma	Beaman et al., 1976
L-form	UPEC	Recurrent UTI	Mickiewicz et al., 2019
L-form	*Enterococcus faecalis*	Chronic bacteriuria	Gutman et al., 1965
L-form	*Klebsiella* spp.	Chronic bacteriuria	Gutman et al., 1965
Small granule-like forms	*Corynebacterium*	Subacute endocarditis	Wittler et al., 1960
CWD	*Mycobacterium tuberculosis*	Sarcoidosis	Almenoff et al., 1996
CWD	*Mycobacteria*	Tuberculous meningitis	Alexander-Jackson, 1945

*UPEC, urinary pathogenic E. coli; UTI, urinary tract infection; CWD, cell wall-deficient.*

The finding of CWDB in persistence and chronic infection provide potential therapeutic approaches to eradicate persisters and cure the chronic infections. Using a combination of agents targeting cell membrane and β-lactam antibiotics, the efficacy of bacterial killing was significantly enhanced (Zou et al., 2020). Similarly, synergistic effects of macrolide antibiotics and cell wall targeting antibiotics was also observed to eradicate the L-form cells of *P. aeruginosa* (Kasai et al., 1982). Cell wall deficient forms of *B. subtilis* were demonstrated hypersensitive to daptomycin and nisin, both of which target the cytoplasmic membrane, indicating a potential therapeutic approach to combine antibiotics targeting cell wall and cell membrane to combat persistence infections (Wolf et al., 2012).

## Conclusion Remarks

Bacterial persistence has recently been recognized as the one of the major causes of infection relapses and emergence of antibiotic resistance. Understanding the molecular mechanisms of persistence formation may provide important implications for the development of novel drugs/approaches targeting the latent persisters. Dormancy has been well-known as the fundamental mechanism of persistence formation. However, recent findings suggest that bacteria may employ diverse strategies for persistence. Other than entering the dormancy to overcome the antibiotic slaughter, bacteria could actively battle with antibiotics for persistence. Further studies are needed to further understand the molecular mechanism of persisters and find solutions to control their infections.

## Author Contributions

JZh initiated the concept. JZo, BP, JQ, and JZh wrote the manuscript. All authors contributed to the article and approved the submitted version.

## Conflict of Interest

The authors declare that the research was conducted in the absence of any commercial or financial relationships that could be construed as a potential conflict of interest.

## Publisher’s Note

All claims expressed in this article are solely those of the authors and do not necessarily represent those of their affiliated organizations, or those of the publisher, the editors and the reviewers. Any product that may be evaluated in this article, or claim that may be made by its manufacturer, is not guaranteed or endorsed by the publisher.
